# Notes of an European Tour

**Published:** 1846-05-01

**Authors:** F. H. Hamilton

**Affiliations:** Prof. of Surgery in Geneva Medical College


### BUFFALO MEDICAL JOURNAL

**Vol. 1.** MAY 1, 1846. **No. 12.**

### Notes of an European Tour.---No. 12.

Communicated for the Buffalo Medical Journal, by F. H. HAMILTON, M. D, Professor of Surgery in Geneva Medical College.

Naples has a population variously estimated from 350 to 450,000, crowded into the narrowest possible space between the high grounds and the sea—dwelling in narrow and irregular streets—occupying every square foot of room from the cellar to the attic of five and six storied houses; and the 40,000 Lazzaroni living on the sands of the beach, and sleeping at night under the sheds of the market places,—beneath the stone gateways and within the cortiles of public buildings! A huge, compact mass of human beings, drawn together, no one can say for what purpose, sustained, no one knows by what means—and who all their lives do little else than laugh and chatter, eat maccaroni, drink wine and say mass. The king—"king of the Lazzaroni," loves his people, as a wolf loves the tender kid. He has a palace in the heart of the city, that he may enjoy their constant proximity; and an impregnable castle directly behind it, to which he may retire when their company becomes irksome. When the cholera decimates the population, he celebrates mass for them in his palace at Portici, and sends word that no one must leave the city; but commands that they confess their sins and renew their vows and their *offerings* at the chapels of their saints. When perishing with famine, they press on him along the Ponte della Maddalena, and throw their black bread into his carriage, he turns his eyes upwards toward the statue of St. Gennaro, and implores a blessing upon the wretches, then orders his secretary to raise the impost upon salt to $3,00 per bushel, and forbids the use of the sea-brine under penalty of the galleys—to levy a new tax for the building of public roads, but which in three years will be legally forfeited to the crown, because the crown has not authorized the disbursement of the sums thus raised, and to enforce rigidly the payment of one-fourth of all the rents into his own exchequer. To-day I saw the son of the King riding along the chiaja and throwing a few grana to the beggars. My friend *whispered* that he was "making a short loan, and he would call for it in a few days with tremendous usury"—that "such liberality always betokened a new and grinding impost." I am not a seer or this youth is the last of that despotic and bigoted house who shall press the iron upon Neapolitan necks; for when, driven by the tempest of the French revolution, the seeds of Liberty were scattered every where over the plains of Europe, a few withered and straggling grains were let fall along the fertile banks of the Sebeto and upon the warm hill sides of Parthenope, where, swelling into life they soon struck their vigorous roots deep and strong, and to this day these plants of liberty defy the power of the "Holy Alliance" to tear them up. The "Jacobins" of 1794 and the "Carbonara" of 1820, were from the same stock with the noble patriots, who a few days since, were thrust into the dungeons of the Vicaria for having dreampt of freedom, and who are to be tried and *executed* upon the testimony of letters surreptitiously obtained from the London Post Office by order of Queen Victoria! an act of comtemptible espionage and royal theft, which "purple" cannot cover.

From narrow and putrid lanes—from taxation, meagre diet and famine—from poverty, rags and homeless beggary—from mental enslavement, political servitude and chains, the transition to Hospitals, Alms Houses, Foundling Establishments, Lunatic Asylums, Prisons and Galleys, is not forced. The one are the fountains and head streams—the other, the artificial pools into which these various tributaries pour their muddy tides.

The "Spedale degl'Incurabili," which I visited in company with an English Physician for many years a resident of Naples, is situated in a very pleasant and sufficiently elevated part of the city, not far from the College of Medicine and Surgery, and the famous "Museo Borbonico."

It was established and endowed by a Lady of fortune in 1519, and having since then received frequent munificent donations, it has been from time to time enlarged, until now it is the most extensive hospital in the city, and will accomodate more than 1200 patients; who, as the name of the hospital indicates, are generally afflicted with chronic and "incurable" diseases. Females, however, suffering from lues, whether acute or chronic, are sent to the Hospital "Di La Fede"—there is also at the S. d. Incurabili a ward for lying-in women, but devoted exclusively to the unmarried. The infant may be retained by the mother when she leaves, or it may be sent to the Foundling Hospital, to be forever excluded from a knowledge of its parentage. As a suffix to this latter ward, there is a portion of the building allotted to such females as wish to retire from the world.

Connected with the hospital are four Clinical chairs, viz. Medicine, Surgery, Obstetrics and Opthalmology. There is also a chair of practical Anatomy, which was formerly occupied by the celebrated Dominicus Cotugno ("Cotunnius") whose name is familiar to the Student of Anatomy in connection with the vestibule of the ear, and the spheno-nasal branch of the superior maxillary nerve. Cotugno died in 1822, having bequeathed to this hospital 80,000 ducats, or about $64,000 In a large and sombre apartment—the "the Consultation Room"—among a number of portraits hangs that of Cotugno, distinguished for its mild but intellectual countenace.

The wards of this hospital are spacious and well ventilated, but not remarkable for neatness or the order of internal arrangements. I missed here for the first time in Europe the "Sisters of Charity"—their places being supplied by male attendants—and perhaps this is sufficient to account for a more than usual lack of cleanliness. Possibly my not meeting with any of the "Sisters" might have been merely accidental. The two wards devoted to phthisical patients did not appear comfortable. The ceilings are arched and rather low, and the windows arranged upon only one side of the appartments, are too loose and open to secure the occupants from the damp and chilling tramontane. The number of consumptive patients is now about 100, and of all the inmates they seemed the least cared for. In all the South of Italy, consumption is believed to be contagious; at least by the people; and I am told that many respectable physicians hold the same opinion. The consequence is that most of the foreigners suffering under this or any similar malady are driven from both private and public houses to the hospitals, where they are lodged as in a "Pest House"—entirely separated from the other wards, and avoided as much as possible by the "Capi Corsee," or ward servants. The floor was dirty, the linen soiled and the patients, each of whom administered to himself from a cup of antimonial solution, looked sad and friendless, and altogether it was a most sorrowful place.

In a small room, called the Museum, may be seen with other anatomical and pathological specimens, a part of the cabinet of the celebrated Scarpa.

The "Spedale della Missericordiella" situated not from the "S. d. Incurabilli" I did not visit. I understood however that it belongs to that very humane society the "Misericordia," whose members are occasionally seen in the streets, enveloped in a complete mask; of which fraternity I shall hereafter take occasion to speak and to describe more particularly. The order exists throughout Italy, and I shall doubtless meet with them again, and learn more of their character, duties, &c.

"Santa Maria della Fede" is still beyond the "Misericordiella'—in a quarter of the town to which "Dickens Points" of New York, St. Giles of London, or La Cite of Paris afford no parallel—for here is series of streets, forming a city in themselves, where every house is a lupanare, every man a pander and every woman a courtezan. The hospital of "S. M. d. Fede" is devoted exclusively to these wretches, and corresponds to La Loursine of Paris. The civil and medical police regulating this class of citizens at Naples is similar to that of Paris, but neither as rigid, systematic, or effective. Such open violations of decency are not witnessed, I am confident, in any other city in the world, as in these horrible purlieus.

Returning again westward, toward the centre of the city, we see not far from the "Vicaria", or state prison, a dark prison-looking building, situated upon the Piazza Capuana. This is the "S. San Francesco", or hospital for the reception of sick convicts. Passing by which, for you will not be admitted except by special permission, and crossing the court containing the prison and courts of justice, you enter the "Strada de Tribunali," on the east of which stands the "Spedale della Pace"—occupied by males affected with fevers, inflammations, &c., and which is at the same time one of the smallest and neatest charities in the city.

To see the hospital ("Santa Eligio") devoted to females laboring under similar diseases, you must leave this comparatively wholesome portion of the city and thread your way southward a long distance, among irregular and constantly darkening streets, untill you find the "Piazza del Mercato," the principal market square; celebrated in Neapolitan history as the spot where in 1647 Thomas Anniello, by the populace called Masaniello, commenced his famous revolt, and upon which in 1268 the young Prince Conradin was executed. The hospital stands just off from this square, amid loathsome odors and a more loathsome population. How the poor sick females can manage to live in spite of the braying of jacks, the shouting of market men, the malaria, &c. I cannot conceive, unless indeed, the inmates were all residents of this quarter, and had thus become acclimated before they were received.

The hospital of "Loreto" is about half a mile out of the Porta Carmina on the road to Vesuvius.

The hospital of the "Pellegrini" is in quite the opposite part of the city, and not far distant from the spacious piazza called the "Santo Spirito". In it are admitted male patients who have received surgical injuries. The number is not large, but the building is ample and commodious.

There are in the city two principal military hospitals for the army, "La Trinita," and "La Sagramento." "La Trinita" is on the slope of the hill upon which is placed the formidable castle of St. Elmo. The building was originally a convent, and is one of the thousands which Napoleon, (in this instance *Murat*,) suppressed in Italy, and with the lands and revenues devoted to a *useful* purpose. This convent, Murat gave up to his brave soldiers, of whose comfort he was never less mindful than his brother; — for the monks of La Trinita had chosen one of the most delightful and airy spots in the city, and their green and well shaded terrace commanded a complete view of Naples and the bay, from whence, now, such of the patients of the hospital as can see, for a large number have lost their sight, enjoy daily the splendid panorama spread beneath them. With such a residence one might almost envy them their misfortunes.

When Gualandi of Bologna, wrote of this hospital in 1823, he noticed the great prevalence of opthalmia among the soldiers of the Italian army, and he ascribed it to the "fine dust formed from the tufa rock upon which Naples and all the adjacent country stands; to the use of stimulating drinks;" (principally, but not entirely the native wines,) "to chilly nights succeeding hot days; to the reflection of the sun and to contagion." But since opthalmia is not especially prevalent among the citizens of Naples, (indeed I have not seen a blear eye, and rarely a blind eye among all the mendicants, those intinerating lazars of Italy,) who are equally exposed to most of the above assigned causes — and having noticed that a large number of the soldiers were under treatment in the syphilitic wards, I have thought that to gonorrhœa and lues might be ascribed many of those destructive opthalmic inflammations. The hospital contains not far from 900 patients.

"La Sagramento" is smaller and not as pleasantly situated, but in the same quarter of the city.

Following me in my rambles back to the "chiaja," the navy and marine hospital called Piedigrotta, may be visited while you are paying a visit to the Tomb of Virgil and the "Grotto of Posillipo," near the eastern entrance of which it stands. The wards are more like those of American Hospitals than any I have yet seen, being smaller and more numerous, and thus effecting a more complete separation of the patients: at the same time that by the arrangement of windows and doors "vis a vis" a perfect ventilation is ensured. No one feature of nearly all the European Hospitals have I felt so much disposed to censure as the great extent of the "salles" and the consequently large number of invalids who are crowded into one apartment within sight and within sound of each other, and who are compelled thus to hear the groans and crazy mutterings of their dying fellow sufferers, and to witness perhaps the ill suppressed agonies of the only friend, a wife perchance or a husband, who has obtained permission to be present at this last hour. Here a priest with incense and holy water is administering "extreme unction," there the rude servants are closing the eyes and straightening out the limbs, scarcely waiting for the breath to cease from the lips. Such scenes as these are of daily occurrence, and who can doubt but that their influence upon the unfortunate survivors is sadly depressing, to say nothing of the effect of the noise, confusion and bad air inseparable from the presence of such numbers.

Such a charity, as the "Massachusetts General Hospital" I have not yet met; I mean in point of comfort and in excellence of internal arrangement. The hospital of "Piedigrotta," is however, the nearest approach. The galley slaves who work in and about the arsenal, when sick or injured are sent here also.

A few rods from my door, fronting also upon the bay, is the Royal hospital for the blind, established in 1818, chiefly through the exertions of Senore Quadri.

Naples has also a large number of Hospices; the principal of which is the "Seraglio" or "Albergo dei Poveri," situated without the city on the broad and beautiful road, which leads to modern Capua. This is an establishment which deserves notice. The building is yet incomplete, it being proposed that the facade of each of its four sides shall be 1600 feet, and the whole sufficiently large to accomodate 10,000 persons. At present the principal front is over 1000 feet in length, four stories high, and built of stone. Within are three large courts, (when complete there will be five) the entrance is through a beautiful portico, with three arches; the centre arch opening into the church: one of the side arches leading to the apartments of the females and the other to that of the males. About 5550 persons, including orphans, are now sheltered, fed, clothed and *instructed*, in this establishment. The females are taught to sew, knit, work coral, weave linen fabrics, and spin with the distaff. The males are taught various mechanical arts; also to read, write, compute, draw, engrave, &c., &c. A small number are sent regularly to the Hospitals and the University, to receive instruction as "Surgeons." (Not Physicians—to introduce paupers into the fraternity of physicians might offend; but surgeons rank here, as in Palermo, with perruquiers and barbers!) A certain proportion are also educated for the Priesthood; but by far the largest number are trained for the army, the whole of the able bodied male lads being subjected to a regular military discipline and drill. The same rule with regard to the girls obtains here as in the A. d. Poveri, at Genoa, if they marry out of the house they receive 30 ducats, equal to about $24; which to us might seem a pitiful sum, but to a Neapolitan beggar it is enough for food, lodging and raiment, such as they usually get, for a year. One third of all the labor of the inmates belongs to themselves, and the balance to the government, which last amount, together with the income from the property belonging to the house, furnishes a revenue of 200,000 ducats, to which also the government is pledged to add annually 40,000 ducats.

When this building is completed, it will be the largest hospital in the world, and will probably contain the largest number of paupers, for if it was five times as large it could be filled, and the ranks of the paupers not be sensibly thinned in the kingdom of Naples. The inmates being drawn not from the city of Naples only, but as the inscription over the main entrance implies, from the whole kingdom. But it is very much to be doubted whether, with such a crowded population, however perfect their means of ventilation, the air can be kept sufficiently pure for health, and whether if any contagious or highly pestilential disease should gain admission, it would not become at once a vast lazar house, which it would be dreadful even to approach.

Adjoining the hospital, toward the city, is a large and flourishing botanic garden, handsomely laid out in squares and adorned with fountains. At present it is under the direction of Signore Michael Tenore, author of the "Flora Neapolitana," and a botanist of high rank. It is open to the public daily, and at this season of the year it is a pleasant and fashionable promenade.

